# 

**Published:** 1881-05

**Authors:** 


### CONTENTS:

| | | Page. |
|---|---|---|
| ***ORIGINAL.*** | | |
| Perverted: The History of Homœopathy, | Editorial, | 167 |
| Fatal Errors, | Ad. Lippe, M.D., Phila. | 171 |
| Intermittent Fever, with cases, | Geo. H. Clarke, M. D., Phila. | 173 |
| Action of Homœopathic Globules. | Dr. Malin, | 178 |
| Endorsement, | Editor, | 181 |
| The *Observer*, | "International." | 184 |
| Hæmorrhoids and their treatment, | Dr. Charge, Paris, | 188 |
| ***CLINICAL CASES:*** | | |
| *Apis*—Tonsilitis, *Lach*—Sore throat, W. A. Hawley. *Lilium tig*—Palpitation, *Carbo veg.*—Dysentery, *Sulph.*—Diarrhoea, *Cauth.*—Urinary disease, with comments, | C. Pearson, | 195 |
| Veterinary Cases: *Lyc*—Pneumonia, D. Wilson; *Nux*—Cerebro-Spinal meningitis, E. Bayard; *Graph*—Hoof disease in the horse, | R. R. Gregg, | 201 |
| Periscope, | | 211 |
| ***NOTICES, COMMENTS, &c.*** | | 222 |

